# 

**Published:** 1809-06

**Authors:** 


### NEW MEDICAL PUBLICATIONS.

The Annual Medical Register, comprising a Review of every Publication relating to Medicine and Surgery, which appeared in 1808; with a Sketch of the Discoveries and Improvements in those Sciences, &c. By a Society of Physicians, 8vo. 9s.

A System of Operative Surgery, founded on the Basis of Anatomy. By Charles Bell, vol. 2, royal 8vo. 16s.

An Inquiry into the Symptoms and Treatment of Carditis, or the Inflammation of the Heart, illustrated by Cases and Dissections. By John Ford Davis, M.D. 12mo. 6s.

Medico-Chirurgical Transactions, Vol. I. illustrated with ten plates, 14s. boards.

Thomæ Simsoni Medicinæ Professoris Candossensis in Academia Andreana, apud Scotos, De Re Medica, dissertationes quatuor. In usum Medicinæ et Humanitatis Studiosorum iterum excudi curabat. Andreas Duncan, senior, M.D. et P. Principis Scotiæ Medicus Primarius. 7s. 6d.

### CORRESPONDENCE.

Dr. FERGUSON, in a letter to the Editors, dated Aberdeen, May 18, 1809, writes as follows: "I am obliged to you for your suggestion; whether my complaint was occasioned by putrid or specific effluvia, the effect was instantaneous, and the disease had all the distinguishing symptoms of Scarlatina, such as affection of the throat, efflorescence, desquamation, and anasarcous state of the legs. But what is a stronger confirmation, although I have attended many cases of this disease when it has been raging as an epidemic, I never was again affected with it. The disease, however, was not communicated to any other person, as very few had access to me when I was ill." —— "In my paper on Measles, I observe two typographical errors; one at page 362, which should read 'a pupil of Dr. Fraser's, instead of Travers;' the other at page 365, last line save one, which should be 'sevum cetæ mixture, instead of cerum cetæ."

We are certain Mr. TAYLOR cannot be aware of the severity of some parts in his Letter; and can only say, as we did to his Correspondent, that if he will permit us to omit the objectionable passages, all that relates to the Controversy shall find admission in our next Number.

END OF VOL. XXI.

### INDEX.

ABDOMEN, cafe of a wound in the 414
Aberdeen, epidemic meafles at 359
Abfcefs, connected with the pericardium, cafe of 265
Abftinence, extraordinary cafes of 60, 96
———, from bodily exercife, on 227
Acarus, obfervations on the 8
Actions, on two difeafed 109
Æther in hydrophobia, on 240
Agaric, obfervations on 13
Ague, virtue of the fpider's web in 353
———, on arfenic in 441
Air, on examining the ftate of the 91
——— effects of fixed 385
Air-baths, improvements in 85
Alder, Dr. on the proximate caufes of difeafes 102, 298, 449
Alkalies, on the metallic bafis of 27
Allen's, Mr. cafe of abftinence 60
Amenorrhœa, obfervations on 31
Anatomy, on the ftudy of 181
——— on the proximate caufes of difeafes from 296
Anchylofis, obfervations on 424
Aneurifm of the femoral artery, cafe of 424
Anguis fragilis, account of the 6
Angus, Mr. on the trial of 336, 426
Animal heat, obfervations on 87
Animals, on cruelty to 335
Antifcorbutic fyrup, preparation of 290
Apoplexy, obfervations on 286
Arnold, Dr. on typhus, dyfentery, and fcurvy 17
——— on the health of failors 19
Arfenic, its ufe in chronic difeafes 149
——— a cure for the bite of ferpents 277
——— on detecting minute portions of 421
——— on its efficacy in agues 441
Artery, cafe of wounded 317
Affafœtida, hiftorical account of 475
Afthma, obfervations on 94
Atmofpherical obfervations 437
Atrophy in children, remedy for 13
Authors, on a fraud of 412

Bacon, Lord, character of 2
Barlow, Mr. on the origin of calculi 148
Barometer, ftate of the 436
Barzellotti, Mr. on mufcular contraction 457
Bath hofpital, cautions recommended at the 526
Baths, improvements in 85
Beddoes, Dr. vindication of 89
——— memoirs of 183
——— on digitalis 329
Bellamy's, Dr. cafe of lacerated urethra 209
Bengal, diftempers of 75
Bile, to determine the capacity of 69
Blair, Mr. queries by 438
——— reply to 478
Bleeding, its efficacy in difficult parturition 179
Blindnefs, on hereditary 232
Boat, account of a new life 173
Book-making, on medical 412
Books, inquiry concerning medical 240
BOOKS, CRITICAL ANALYSIS OF NEW MEDICAL — Effay on Difeafes incidental to Europeans in hot Climates, by J. Lind, M. D. 71. Practical Obfervations on Strictures of the Urethra, by W. Wadd, 79, 161. The Edinburgh Medical and Surgical Journal, No. XVII. 146. Obfervations on the Egyptian Ophthalmia, by W. Thomas, 164. A Letter to the Bifhop of Durham, propofing a plan for improving Difpenfaries, &c. by J. Herdman, M. D. 166. New Medical Compendium for the Ufe of Families, by D. Cox, 167. A Syllabus of a Courfe of Lectures on the Medical Sciences, by T. Young, M. D. 168. A Letter to J. Haygarth, M. D. on Peftilential Fever, by C. Chifholm, M. D. 241. Obfervations on an Eruptive Difeafe, by R. Pew, M. D. 250. *Verfuch einer Prufung und Verbefferung der jetz gewohnlichen Behandlungfart des Scharlach Fiebers, von* Dr. Johann Hieglitz, 252. Trial of Charles Angus for Murder, with the fubfequent Pamphlets, 336, 426. Edinburgh Medical and Surgical Journal, No. XVIII. 414. Obfervations on Madnefs and Melancholy, including Practical Remarks on thofe Difeafes, by J. Haflam, 495. Reports on the Effects of a peculiar Regimen on Schirrous Tumours and Cancerous Ulcers, by W. Lambe, M. D. 508
BOOKS, NEW, ANNOUNCED—Dr. Herdman on the Improvement of Difpenfaries, 86. Dr. Kidd's Outlines of Mineralogy, *ib* Reports of the Medical and Chirurgical Society of London, *ib*

Mr Arnold on the Management of the Infane, *ib.* Dr. Adams on the Venereal Difeafe, 175, 440. Dr. Kentifh on Warm and Vapour Baths, 176. Dr. Stock's Life of Dr. Beddoes, *ib.* Reports of the Preventive Medical Inftitution at Briftol, *ib.* Mr. Macartney on the Relation between the External and Internal Parts, *ib.* The Annual Medical Regifter, 263. Mr. Saunders on Difeafes of the Eye, *ib.* Mr. Martin on the Petrifactions of Derbyfhire, 351. Mr. Ruffell's Syftem of Surgery, *ib.* Dr. Thomfon's Syftem of Surgery, *ib.* Mr. Maryan on Hydrophobia, 440. Mr. Wilfon's Pharmacopea Chirurgica, 527. Dr. Serny's Treatife on local Inflammation, 528
Borrett's, Mr. cafe of hydrophobia 268
Boftock's, Dr. experiments on poifon 148
——— on detecting arsenic 421
Bougie, account of the armed 81
——— obfervations on the 309
Bowels, cafe of conftipation of the 480
Brain, on the anatomy of the 149
Brazil, vaccine inoculation in 173
Bree, Dr. on the caufe of purpura 321
Briggs's, Dr. cafe of tetanus 418
Britain, on a medical typography of 1, 91
Brown's, Dr. cafe of conftipation 480
———, Mr. cafe of difficult parturition 179
Browne's, Mr. cafe of wounded artery 317
Bryonia dioica, obfervations on the 12
Burns, ufe of ftimulants in 386

Calculi, on the origin of 148
——— extracted from the bladder of a dog 369
Calomel, various ufes of 118
Cancer, obfervations on 508
Candour recommended 121
Cantharides, efficacy of in gleets 519
——— on the free ufe of 49
Carlifle, Mr. on mufcular motion, remarks on 457
Carmichael, Mr. on anchylofis 424
Cataract, on the extraction of the 148
Catarrh, prevalence of 259, 333
Cauftic in ftrictures, obfervations on 79
Ceylon, vaccination in 126
Charcoal and hydrogen, obfervations on 527
Children, on a difeafe affecting the hair of 228
——— on the different capacities of 230, *note*
Chinefe method of raifing fruit-trees 86
Chifholm, Dr. on peftilential fever 241
Chriftie, Mr. on vaccination in Ceylon 126
Chromatometry, account of a lecture on 171
Clarke's, Dr. medical reports 425
Climates, on the difeafes of hot 71
Cocinella feptem punctata, great increafe of the 9
Colica pictonum, cafe refembling the 406
Conftipation from cold, cafe of 406
Confumption fuccefsfully treated with mercury 117
Contagion, obfervations on 241
Contraction, on mufcular 457
Convulfions from the poifon of a rabid animal, cure of 140
Cornea, on making an incifion of the 146
Correfpondence 88, 176, 264, 352, 528
Cranium, on the capacity of the 234
Criticifm, on admiffible 89
Croup, on the treatment of 518
Cullen, Dr. on the temperaments 200
Currie, Dr. anecdote of 413
Cynanche tonfillaris, treatment of 82, 170, 433
——— trachealis, on the 97

Daphne laureolæ, efficacy of 13
Darwin, Dr. on the temperaments 199
Davies, Mr. on the vaccine conteft 121
Davy, Profeffor, account of his Galvanic battery 527
———'s Mr. difcoveries vindicated 129
——— anfwer to inquiries 350
Deformities, on hereditary 235
Dentition, on difeafes of 281
Derbyfhire, falubrity of 95
Desfontaine's, M. memoir on jalap 392
Digitalis, on the ufe of 325
Difeafes and cafualties of London 87
——— of the female organs, on the 29
——— in London, reports of 84, 261, 349, 434, 522
——— in Edinburgh 82, 168, 258, 332, 431, 517
——— proximate caufes of 102, 296
——— of hot climates, obfervations on 71
——— obfervations on hereditary 229, 281
Difpenfaries, plan for improving 86, 166
Dogs, on the ill treatment of 335
——— on the calculi in the bladder of 369
Drake, Dr. on digitalis 329
Dreaming, obfervations on 502
Dropfy, obfervations on 285
Dudley, account of a mineral fpring at 439, 464
Durham, letter to the bifhop of 166
Dyfentery, obfervations on 18

Eaft Indies, difeafes of the 74
Edinburgh, reports of difeafes at 82, 168, 258, 332, 431, 517
——— proceedings of the Natural Hiftory Society at 171, 262
——— Journal, account of 146, 414

Egyptian Ophthalmia, obfervations on the 164
Empirics, on the practices of 101
Elephantiafis, on the 287
Epidemic meafles, on 359
Epilepfy, cafe of 239
Eruptive difeafe, obfervations on an 250
Erythema, cafe of 417
Europeans, on the difeafes of in hot climates 71
Experiments on animal heat 67
——— on a calcareous ftone 85
——— on mineral poifons 148
——— on platina 262
——— on potaffium 356
——— for detecting minute portions of arfenic 421
——— on James's powder 438
——— on the mineral fpring at Dudley 439, 464
——— on marble 439
——— on mufcular motion 457

Family difeafes, obfervations on 229, 281
Female organs of generation, difeafes of the 29
Femoral artery, cafe of aneurifm of the 424
Fens, obfervations on the 92
Fergufon, Dr. on the temperaments 198
——— on the epidemic meafles 359
——— letter to the Editors 527
Ferriar, Dr. on digitalis 327
Fevers, on the proximate caufes of 102
——— on the prevalence of typhus 169
——— on peftilential 241
——— virtue of fpider's web in 353
——— obfervations on 433
——— on arfenic in intermittent 441
Fleming's, Mr. defcription of a fea unicorn 171
Flooding, cafe of 424
Flora, on the formation of a medical 9
Forceps, on the ufe of the 382
Fourcroy's, Mr. analyfis of calculi 369
Fruit-trees, Chinefe method of grafting 86

Gall, Dr. report on a memoir of 149
Galvanic battery, account of 527
Galvanic combinations, on the 350
Garat, remarkable anecdote of the family of 229
Generation, on difeafes of the organs of 29
Glaziers, &c. cautions to 526
Gleet cured by cantharides 519
Gliffon, Dr. on mufcular motion 459
Gout, obfervations on 285
——— cafe of 425
Grafting, Chinefe method of 86
Great Britain, on the medical topography of 1, 91
Green ftone, defcription of the 171
Gregory, Dr. anecdotes of 234
——— on fevers 450
Grenada, on the fever of 241
Guinea, defcription of the coaft of 73

Hæmatocele, obfervations on 389
Hague, Mr. on a wound in the abdomen 414
Hall, Mr. on a difeafe in the hair of children 228
Hamilton, Dr. on hydrophobia 272
——— on digitalis 330
Hardwick, Mr. on hydrophobia 23
Harrup, Mr. on two difeafed actions 109
Haslam, Mr. on madnefs 495
Head, on the bulk and form of the 334
Heart, on hereditary palpitations of the 237
Heat, obfervations on animal 67
Hedera helix, properties of the leaves of 13
Hemlock, on the properties of 11
Herdman, Dr. on difpenfaries 86, 166
Hereditary difeafes, on 229, 281
Hernia, on the prevalence of 97
Herniary fac, hydatids in a 278
Hieglitz, Dr. on fcarlatina 252
Hill, Mr. on the ufe of arfenic 149
——— on the Liverpool controverfy 399
Hoffack's, Dr. cafe of aneurifm 424
Howfhip's, Mr. cafes of locked jaw 180, 407
——— cafe of local inflammation, &c. 265
——— on the treatment of tetanus 446
Hunter, Mr. on a doctrine of 109
Hydatids, cafe of obftruction from 225
——— in a herniary fac 278
Hydrocephalus internus, treatment of 83
Hydrocephalus, on 259
Hydrophobia, Mr. Hardwick on 23
——— Dr. Pinckard's cafe of 53
——— Dr. Wood's obfervations on 134
——— Mr. Taunton on 219
——— queries on 240
——— Mr. Borrett's cafe of 268
——— Dr. Roberton on 334, 519
Hydrops medullæ fpinalis, cafe of 66
Hydrothorax, cafe of 443
Hyfteria, virtue of affafœtida in 477

Idiofyncrafy, cafe of 486
Incifion of the cornea, on making the 146
Infants, on the treatment of 83
——— on the rofes of 334
Inflammation, cafe of local 265
Infanity, obfervations on 100
Intelligence, mifcellaneous 85, 170, 262, 350, 438, 526
Intermittents, on the caufes of 92

Intermittents, virtue of fpider's web in 353
——— on arfenic in 441
Ipecacuanha, fingular affection from 486
Iffues in paralyfis of the lower extremities, remarks on 109
Jacobus on alkalies 27
——— anfwers to 129, 264
Jackfon, Dr. on the virtues of the fpider's web 353
Jalap, memoir on 392
James's powder, analyfis of 438
Jenkinfon, Mr. on abftinence from exercife 227
Johnfon, Dr. anecdote of 501
Jurifprudence, on medical 100

Kenworthy's, Mr. cafe of obftruction in the urethra 225
Kidd's, Dr. outlines of mineralogy 86
Kinglake, Dr. on criticifm 89
——— on phthifis pulmonalis 193

Lambe, Dr. on fcirrhous tumours 508
Laurence's, Mr. obfervations on lithotomy 415
Lead, cautions to thofe who work in 526
Lectures, medical announced.—Dr. Buxton, on the Theory and Practice of Medicine, 87. Mr. Brookes, on Anatomy, Phyfiology, and Surgery, 88. Dr. and Mr. Clarke, on Midwifery, &c. 88, 263. Dr. Clough, on Puerperal Anatomy, Phyfiology, and Pathology, *ib.* Dr. Reid, on the Theory and Practice of Medicine, 88, 263. Dr. Squire, on the Theory and Practice of Midwifery, *ib.* 351. Mr. Taunton, on Anatomy and Surgery, 263, 527. At St. Thomas's and Guy's Hofpitals, 175. Mr. Carpue, on Anatomy, Surgery, &c. *ib.* 440. Mr. Chevalier, on the Principles and Operations of Surgery, *ib.* Dr. Clutterbuck, on the Theory and Practice of Phyfic, *ib.* Drs. Dennifon, on the Theory and Practice of Midwifery, *ib.* Dr. Hooper, on the Theory and Practice of Phyfic, *ib.* Dr. Ramfbotham, on the Science and Practice of Midwifery, 263, 351
Leucorrhœa, cafe of 39
Library, inquiry concerning a 240
Life-boat, defcription of a new 173
Lind, Dr. on the difeafes of hot climates 71
Lithotomy, obfervations on 415
Liverpool controverfy, on the 399, 426
Local peculiarities, obfervations on 5
Lock jaw, cafes of 180, 407
London, reports of difeafes in 84, 261, 349, 434, 522
London, difeafes and cafualties in 87
London, anniverfary meeting of the medical fociety of 350
Longevity, obfervations on 15, 96
Lumbago in women, on the 32

M'Gregor, Dr. on peftilential fever 241
Maclean, Dr. on digitalis 330
Madnefs, obfervations on 495
Magnefia vitriolata recommended in fcarlatina 256
Maniacs, of the brain in 339
Marble, experiments on 439
Marrubrium, virtues of the 13
Marfh miafma, on the 92
Meafles, on the epidemic of 1808 359
Medical topography of Great Britain, on the 1, 91
——— jurifprudence, remarks on 100
——— Society, meeting of the London 350
——— library, inquiry concerning a 240
——— book making, on 412
Melancholy, obfervations on religious 507
Menorrhagia, on the caufes and treatment of 31
Menftruation, obfervations on 30
Mercury, efficacy of in fcarlatina 257
———, its fuccefs in confumption 117 *note*
———, inefficacy of in tetanus 446
Merriman's, Mr. cafe of inverted uterus 63
Michaux, M. on jalap 398
Mineral bafes of alkalies, on the 27
——— fpring, account of a 439, 464
Mineralogy, outlines of 86
Moon, influence of the, on difeafes 75
Moore, Anne, remarkable cafe of 61
Mortality, Dr. Spalding's bills of 523
Morbid anatomy, on the phrafe 89
Morrifon's, Mr. cafe of tinea capitis 177
Mofchus, its ufe in fcarlatina 257
Moffman's, Dr. reply to Mr. Taylor 325
Moucho-more, remarkable qualities of the 14
Mufcle, on the poifon of the 8
Mufcular contraction, on 457

Narwhal, defcription of the 171
Natural Hiftory of Edinburgh, proceedings of the 171, 262
New, Dr. on charcoal and hydrogen 527
Nofe, curious obfervations on the 234
Nottingham, medical reports for 425

Ogilvie's, Dr. mineralogical report 171
Oldknow's, Mr. cafe of ulcer 422
Ophthalmia, obfervations on 164
Opium, on the production of 10
——— its ufe in rabies canina 25
——— the effects of in fcarlatina 257
Organs, on the difeafes of the female 29

Pancras, report of the small-pox hospital at 172

Paralysis of the lower extremities, on issues in 109

Paracelsus, character of 1

Paris, Dr. on animal heat 67

Parkinson, Mr. on several species of marble 439

Parry, Dr. on venesection 146

Parturition, case of difficult 179

Pathological anatomy, on the phrase 89

Pericardium, local inflammation and abscess connected with the 265

Pestilential fever, observations on 241

Pew, Dr. on an eruptive disease 256

Phthisis pulmonalis, observations on 193

——, on abstinence from exercise in 227

——, observations on hereditary 281

——, virtue of spider's web in 358

——, prevalence of, at Nottingham 425

Pick's, Mr. analysis of a calcareous stone 85

Pinckard's, Dr. case of hydrophobia 53

Pitcairn, Dr. memoirs of 489

Plague, on the 106

Platina, on the elasticity of 262

Pneumonia, on the treatment of 83

Poison, experiments and observations on mineral 148

Pole's, Dr. thermometrical statements 435

Poor, on the medical treatment of the 166

Poppy, on the cultivation of the 10

Porrigo, on a species of 132

Portal, M. on hereditary diseases 229, 281

Portsmouth (America) bills of mortality at 523

Potassium, on its production by chemical combinations 350

Publications, new medical 88, 176, 263, 352, 440, 528

Pully's, M. analysis of James's Powder 438

Purgatives, case of tetanus cured by 418

Purpura, on venesection in 146

——, remarks on the cause of 321

Quackery, remarks on 100
Queries by Mr. Blair 438

Rabies canina, observations on 219

——, virtues of opium in 25

——, spider's web recommended in 358

Rachitis, observations on 238

Reform, observations on medical 99

Rheumatism, on the treatment of 518

Roberton, Dr. on female diseases 29

—— on the free use of cantharides 49

—— report of diseases at Edinburgh 82, 168, 258, 332, 431, 517

Roberton, Dr. on strictures in the urethra 303

—— on permanent stricture 371

—— on hydrophobia 354, 519

Robertson's, Mr. case of flooding 424

Rot in sheep, on the 93

Royston, Mr. on a medical topography 1, 91

Rutter's, Dr. case of erythema 417

Sailors, on the health of 19

Scald-head, on the 328

Scalp, species of porrigo affecting the 132

Scarlatina, essay on 252

Scott, Mr. on the elasticity of platina 262

—— on wounding the septum scroti 389

—— his case of hydrothorax 443

Scrophulous diseases, remedy for 464

—— observations on 286

Scurvy, observations on 17

Septum scroti, on wounding the 389

Sheep, on the rot in 93

Skin, observations on spots on the 230

Sleep, observations on 500

Small-pox hospital, report of the 172

Snake, observations on the sea 262

Sowerby's, Mr. lecture on chromatometry 171

Spalding's, Dr. bills of mortality 523

Spasmodic diseases, effects of tobacco in 27

—— stricture, observations on 306

Spencer's, Mr. case of idiosyncrasy 586

—— observations on lacerated urethra 487

Spider's web in fever, virtues of the 353

Spine, on the curvature of the 109

Spina bifida, case of 65

Spots on the skin, observations on 230

Spring, account of a mineral 439, 464

Stimulants in burns, use of 386

Stone, analysis of a calcareous 85

—— description of a remarkable 171

—— on the relation between the gout and 285

Stricture, remarks on permanent 303, 371

Sulphuric acid recommended in scarlatina 252

Swedish sailors, on the health of 19

Taunton, Mr. on rabies canina 219

—— on hydatids in a herniary sac 278

Taylor, reply to Mr. 325

Temperaments, on the 198

Tetanus, cases of lock jaw with 180, 407

—— cured by purgatives 418

—— on the treatment of 446

Thermometrical statements 435

Thomas, Mr. on the ophthalmia 164

—— Dr. on digitalis 330

Thunder-pick, analysis of a stone called 85

Tides, influence of the 75

Tinea capitis, case of 177

——, effect of fixed air in 385

Tobacco, its effects in spasmodic diseases 27

Topography, hints for a medical, 1, 91

Trees, Chinese method of raising fruit 86

Tumours, on the treatment of scirrhous 508

Tygers of Africa, remarkable ferocity of 73

Typhus, observations on 17

—— prevalence of 84, 169

Tympanites cured by assafœtida 477

Udiometrical observations 435

Ulcer, case of an old 422

Ulcers, on the treatment of scirrhous 538

Underwood, Dr. on paralysis 109

Unicorn, description of a sea 171

Urethra, case of contused 367

——, on strictures of the 79, 161

——, case of lacerated 209

——, case of obstruction in the 225

——, remarks on strictures in the 303, 371

——, on a case of lacerated 487

Uterus, case of inverted 63

Vaccination in Ceylon, account of 126

—— in the Brazils, state of 173

—— on an eruptive disease after 250

—— Mr. Blair's queries on 438

Vaccine contest, on candour in the 121

—— inoculation, queries concerning the 438

—— establishment, account of the national 170

Vapour bath, improvements in the 85

Vena saphena, fatal case of tying the 422

Venesection in purpura, observations on 146

Verax, on a case of contused urethra 367

Veterinary art, on the improvement of the 98

Viper, case of the bite of the 7

Wadd, Mr. on strictures in the urethra 79, 161

Wardrop, Mr. on extracting the cataract 146

Washbourn's, Mr. case of hydrops medullæ spinalis 66

Wasp, appearance of a new species of 173

Web, virtues of the spider's 353

Weather, observations on the 82

Weldon, Mr. on a mineral spring 439

Wernerian society, papers read at the 171, 262

White, Mr. on a species of porrigo 132

Withering, Dr. on digitalis 327

Wood, Dr. on hydrophobia 134

Yellow fever, on 242

Young's, Dr. syllabus of lectures 168

### REFERENCES to the PLATES.

PAGE
Mr. Howship's Case of local Inflammation - - - - - - 265
Machine for raising sick or burnt Persons - - - - - - - 386

